# Organoid modeling meets cancers of female reproductive tract

**DOI:** 10.1038/s41420-024-02186-x

**Published:** 2024-09-27

**Authors:** Jiao Li, Mengting Zhou, Jun Xie, Jiani Chen, Mengni Yang, Changjun Ye, Shihu Cheng, Miao Liu, Rui Li, Ruirong Tan

**Affiliations:** 1 Translational Chinese Medicine Key Laboratory of Sichuan, Sichuan-Chongqing Joint Key Laboratory of Innovation of New Drugs of Traditional Chinese Medicine, Sichuan Institute for Translational Chinese Medicine, Sichuan Academy of Chinese Medicine Sciences, Chengdu, China; 2 West China School of Pharmacy, Sichuan University, Chengdu, China; 3 School of Pharmacy, Chengdu University of Traditional Chinese Medicine, Chengdu, China; 4 Information Technology Center, West China Hospital of Sichuan University, Sichuan University, Chengdu, China; 5 Chongqing Medical University, Chongqing, China; 6 Rehabilitation Department, Changgeng Yining Hospital, Wenzhou, China; 7 Geriatric Department, Changgeng Yining Hospital, Wenzhou, China; 8 Department of Pathology, Brigham and Women’s Hospital, Harvard Medical School, Boston, MA USA; 9 Department of Radiation Oncology, Radiation Oncology Key Laboratory of Sichuan Province, Sichuan Clinical Research Center for Cancer, Sichuan Cancer Hospital and Institute, Sichuan Cancer Center, Affiliated Cancer Hospital of University of Electronic Science and Technology of China, Chengdu, China

**Keywords:** Gynaecological cancer, Cancer models

## Abstract

Diseases of the female reproductive system, especially malignant tumors, pose a serious threat to women’s health worldwide. One of the key factors limiting research progress in this area is the lack of representative models. Organoid technology, especially tumor organoids, has been increasingly applied in the study of female reproductive system tumors due to their high heterogeneity, close resemblance to the physiological state, easy acquisition and cultivation advantages. They play a significant role in understanding the origin and causes of tumors, drug screening, and personalized treatment and more. This article reviews the organoid models for the female reproductive system, focusing on the cancer research advancements. It discusses the methods for constructing tumor organoids of the female reproductive tract and summarizes the limitations of current research. The aim is to offer a reference for future development and application of these organoid models, contributing to the advancement of anti-tumor drugs and treatment strategies for female reproductive tract cancer patients.

## Facts

- Stem cell-derived organoids function both as models for disease and as tools for studying tissue regeneration. In contrast to 2D cell lines and expensive, time-consuming animal models, organoids more accurately replicate the three-dimensional structure and microenvironment of tumors, preserve tumor heterogeneity and patient-specific genetic profiles, and are well-suited for high-throughput drug screening and personalized medicine research.
- Patient-derived organoids (PDOs) often retain key cellular subgroups from the original tumor, preserving its heterogeneity. This characteristic is crucial for disease modeling, as seen in fallopian tube organoids, which provide insights into the progression of ovarian cancer.
- Organoids are pivotal in high-throughput drug screening and personalized treatment approaches. Ovarian cancer PDOs, for example, have been used to predict patient responses to chemotherapy, such as carboplatin/paclitaxel, and to investigate resistance mechanisms, particularly in HRR-deficient cases.
- Organoids co-cultured with immune cells, such as tumor-infiltrating lymphocytes (TILs), offer a valuable platform for studying immune responses in cancer. In cervical cancer, organoid co-culture with immune cells has shown potential in identifying therapeutic targets and developing immunotherapy strategies.
- Emerging technologies, including CRISPR-Cas9 gene editing, organ-on-a-chip systems, and advanced ECM-based scaffolds, are enhancing the precision of organoid models. These innovations facilitate better replication of the tumor microenvironment, supporting research in gynecological cancers such as cervical, endometrial, and ovarian cancer.

## Open questions

- How can integrating more components of the tumor microenvironment, such as immune cells and fibroblasts, improve the replication of real tumor conditions in organoid models of female reproductive tract cancers?
- What new insights can be gained by co-culturing organoids with microbiota, particularly for understanding endometrial and ovarian cancer development? How can gene-editing technologies like CRISPR-Cas9 further refine organoid models for studying cancer progression and drug resistance?
- What are the key challenges in improving the efficiency of generating organoids from different stages of female reproductive cancers for high-throughput drug screening and personalized treatment?

## Introduction

Despite recent advancements in molecular typing and treatments like targeted therapies and immunotherapy [1, 2], gynecologic cancers affecting organs like the ovaries, uterus, and cervix remain a significant cause of morbidity and mortality in women’s health. This critical challenge underscores the urgent need for more advanced research tools to enhance understanding and improve the treatment of female reproductive tract malignancies.

Currently, commonly used gynecologic tumor models, including in vitro cell cultures and in vivo animal models, each have inherent limitations. Two-dimensional (2D) cell lines are far removed from the biological characteristics of real cells due to their inability to reflect the interactions between different cell subtypes and between cells and the extracellular matrix [3–5]. Animal models are limited by long cycles, high costs, and ethical concerns, which preclude their use in high-throughput screening, and there are shortcomings in reproducing the genetic characteristics of patients’ tumors. Organoids, as efficient and stable ex vivo models that closely resemble native tissue in cellular composition, morphology, function, metabolism, gene expression, and response, offer unique advantages in the study of gynecologic tumors. By constructing patient-derived organoids (PDOs), not only can the development of tumors and their influencing factors be studied, but also drug sensitivity tests can be carried out to guide clinical medication and achieve personalized medicine. This review explores the application of organoids in various diseases of the female reproductive system, with a focus on gynecologic tumors. It also highlights the limitations of current research, aiming to provide a reference for the future development and application of female reproductive tract tumor organoids.

## Cultivation methods and application scenarios of PDOs

Organoids are generated by culturing stem cells with specific growth factors, extracellular matrix (ECM) components, signaling molecules, and nutrients, allowing them to self-organize into miniature, three-dimensional structures resembling organs [6, 7]. During the formation of the female reproductive system and its tumor organoids, different stem cell sources and media components that favor the formation of various organoids are involved in.

### PDOs derived from ASCs or PSCs

Stem cell sources for organoids including pluripotent stem cells (PSCs) such as embryonic stem cells (ESCs) and induced pluripotent stem cells (iPSCs), as well as tissue-specific adult stem cells (ASCs). The methods by which ASCs and PSCs form organoids are distinct. For ASCs-derived organoids, primary tissues from patients are dissociated into functional units containing adult stem cells, or tumor cells are isolated from tumor tissues. These cells are then enriched and cultured in specific three-dimensional media, forming organoids and tumoroids. In contrast, PSCs-derived organoids involve the directed differentiation of embryonic stem cells from human embryonic tissues or induced pluripotent stem cells from adult tissues. This process generates floating cell aggregates, known as spheroids, which are subsequently transferred to an extracellular matrix in specific media to form organoids.

By adding growth factors to the culture medium, mimic the stem cell niche that supports the homeostasis of ASCs, and promotes the division of stem cells while appropriately inhibiting differentiation, ASCs-derived organoids can be constructed. For cancer research, organoids developed from cancer stem cells from patient tumor tissues fall into this category, which are relatively simpler and capable of including various tumor cell subtypes, presenting a more complex genetic background and capturing the patient’s cancer genome to preserve tumor heterogeneity [8]. Consequently, ASCs-derived organoids are extensively used in studies of tumor heterogeneity and personalized drug evaluation due to their ability to reproduce adult tissues well, and have been preserved in the form of biobanks. Besides the establishment of various tumor organoids, including cervical cancer, ovarian cancer, and endometrial cancer, organoids that represent precancerous lesions, such as endometriosis, endometrial hyperplasia [9], and cervical squamous metaplasia [10, 11], have also been developed from patients’ precancerous lesion tissues.

In contrast, organoids derived from ESCs or iPSCs initially possess a more consistent genetic background. Through stepwise induced differentiation, these organoids develop into structures that include more complex cell types, potentially encompassing mesenchymal cells, epithelial cells, and even endothelial cells [12]. However, this process can also lead to the inclusion of cell types that the tissue itself would not normally possess [13]. ESCs involve the use of early embryos, which raises ethical concerns. In 2006, the Yamanaka team first discovered that the introduction of transcription factors OCT4, SOX2, KLF4, c-MYC (supplemented with factors NANOG and LIN28) can reprogram mature somatic cells in mice, termed induced pluripotent stem cells, which have the potential to differentiate into various somatic cells [14–16]. Organoids derived from iPSCs provide a widely applicable model for studying tissue development or disease progression. In the process of generating fallopian tube organoids, iPSCs are directed to differentiate into intermediate mesoderm (IM), the origin of the fallopian tube, by adding CHIR99021, activin A, and BMP4. Further, by modulating Wnt signaling, IM is driven to develop into the Müllerian duct and female reproductive tract rather than the kidneys, and then to promote differentiation into fallopian tube-like precursor cells by using pre-Müllerian growth factor, which eventually develop into fallopian tube organoids with luminal structures over time [17]. In addition to establishing normal fallopian tube organoids using iPSC lines, iPSCs derived from ovarian cancer patients can generate abnormal fallopian tube organoids that carry the same BRCA1 mutations as patients and exhibit cancerous characteristics [18].

### ECM and growth factors help the formation of female reproductive system organoids

The formation and stable culture of organoids require the support of two key exogenous factors: the extracellular matrix and the culture medium. By being embedded into an extracellular-matrix-mimicking scaffold, such as the commonly used Matrigel, which is derived from a viscous protein mixture secreted by Engelbreth-Holm-Swarm mouse sarcoma cells, stem cells can form complex three-dimensional structures using the matrix as a carrier [19]. In addition to using matrix gel as support, organoids can also be cultured using hydrogel matrices [20, 21], chip cultures [22] and even directly in suspension [23]. Given the dense and difficult-to-digest nature of gynecological tumors, a new dual-layer Matrigel organoid culture method can significantly improve success rates and robustly propagate organoids from different stages and subtypes of gynecological tumors [24].

In addition to a few organoids that only need endogenous signals for formation [25], most organoids require exogenous growth factors throughout [26, 27] or at least in the initial phase [28] of the culture process to help cells to self-organize and gradually divide and differentiate along developmental cues similar to organogenesis. In the culture of female reproductive system organoids, commonly added formulations include: ① growth factors that stimulate cell division and differentiation, such as Wnt pathway activators that maintain stemness—Wnt3a and R-spondin1, Noggin that inhibits bone morphogenetic protein (BMP)-driven differentiation, small molecule inhibitors of activin receptor-like kinase-4,5,7 such as A83-01 and p38 inhibitor SB202190; ② hormones such as 17β-estradiol (E2); ③ cytokines such as EGF, FGFs, HGF that promote cell proliferation and N2 supplement that supports neuron growth; ④ other nutritional supplements such as B27 supplement, N-Acetyl-L-cysteine, niacinamide, insulin-transferrin-selenium and so on. In the initial stages of culture, a ROCK inhibitor (Y27632) is often added to prevent stem cell apoptosis. Table 1 lists representative growth factors added to different gynecologic tumor organoid media cocktails, we selected studies that reported a success rate of ≥50% and/or had at least 10 successful reports.

**Table 1:** Medium components for the culture of gynecologic cancers organoids.

	Cervical cancer organoids			Ovarian cancer organoids				Endometrial cancer organoids		
Reference	[50]	[63]	[69]	[39, 41]	[134]	[184]	[119]	[9, 87]	[82]	[95]
Culturing medium	Advanced DMEM/F-12	Advanced DMEM/F-12	Advanced DMEM/F-12^ab^	Advanced DMEM/F-12	Advanced DMEM/F-12	DMEM/F-12	Advanced DMEM/F-12	Advanced DMEM/F-12	DMEM/F-12	Advanced DMEM/F-12
HEPES	10 mM	10 mM	(−)	(−)	10 mM	(−)	10 mM	(+)	10 mM	10 mM
GlutaMax	1x	1x	1x^a^	(−)	1x	(−)	1x	1x	1x	1x
L-glutamine	(−)	(−)	(−)	(−)	(−)	1x	(−)	(+)	(−)	(−)
Noggin	1% CM	100 ng/ml	100 ng/ml^a^ or 40 ng/ml^b^	1% CM	(−)	100 ng/ml	100 ng/ml	100 ng/ml or 10% CM	(−)	100 ng/ml
RSPO1	10% CM	(−)	200 ng/ml^b^	10% CM	(−)	50 ng/ml	100 ng/ml	5% CM	(−)	20% CM
B27 supplement	1x	1×	1x^ab^	1x	1x	1x	1x	1x	1x	1x
Nicotinamide	2.5 mM	2.5 mM	10 mM^ab^	10 mM	5 mM	5 mM	10 mM	5 mM	10 mM	5 mM
n-Acetylcysteine	1.25 mM	1.25 mM	25 mM^ab^	1.25 mM	1 mM	1.25 mM	1.25 mM	1.25 mM	1.25 mM	1.25 mM
A83-01	500 nM	500 nM	500 nM^ab^	500 nM	500 nM	0.25 µM	500 nM	0.25 µM	500 nM	0.25 μM
Forskolin	10 μM	10 μM	10 μM^a^	10 μM	5 μM^c^	(−)	(−)	(−)	(−)	(−)
FGF2	(−)	(−)	5 ng/ml^b^	(−)	(−)	(−)	10 ng/ml	(−)	(−)	(−)
FGF4	(−)	(−)	(−)	(−)	10 ng/ml	(−)	(−)	(−)	(−)	(−)
FGF7	25 ng/ml	200 ng/ml	25 ng/ml^b^	(−)	(−)	(−)	(−)	(−)	(−)	(−)
FGF10	100 ng/ml	(−)	100 ng/ml^a^ or 10 ng/ml^b^	10 ng/ml	10 ng/ml	(−)	10 ng/ml	(−)	(−)	(−)
IGF1	(−)	(−)	(−)	(−)	(−)	20 ng/ml	(−)	40 ng/ml	(−)	40 ng/ml
EGF	(−)	(−)	10 ng/ml^a^ or 50 ng/ml^b^	5 ng/ml	5 ng/ml^c^	50 ng/ml	50 ng/ml	50 ng/ml	(−)	50 ng/ml
HGF	(−)	(−)	(−)	(−)	(−)	10 ng/ml	(−)	20 ng/ml	(−)	20 ng/ml
p38 inhibitor SB202190	1 μM	1 μM	(−)	(−)	0.5 μM	1 μM	10 μM	0.1 μM	3 μM	0.1 μM
ROCK inhibitor	10 μM	(−)	(−)	(−)	(−)	(−)	(−)	(−)	(−)	(−)
N2	(−)	(−)	1x	(−)	(−)	1x	(−)	1x	(−)	1x
Hydrocortisone	(−)	(−)	500 ng/ml^a^	500 ng/ml	500 ng/ml^c^	(−)	(−)	(−)	(−)	(−)
Y-27632	(−)	10 μM	(−)	5 μM	(−)	10 μM^e^	(−)	10 μM^e^	10 μM	10 μM
CHIR 99021	(−)	(−)	3 μM^b^	(−)	(−)	(−)	(−)	(−)	(−)	(−)
Heregulinβ-1	(−)	(−)	(−)	37.5 ng/ml	37.5 ng/ml^c^	(−)	(−)	(−)	(−)	(−)
β-Estradiol	(−)	(−)	(−)	100 nM	100 nM	10 nM	(−)	10 nM	1 nM	2.5 μg/ml
NGR1	(−)	(−)	(−)	(−)	(−)	50 ng/ml	(−)	(−)	(−)	(−)
Prostaglandin E2	(−)	(−)	3 μM^b^	(−)	(−)	(−)	1 μM	(−)	(−)	(−)
Lipid Concentrate	(−)	(−)	(−)	(−)	(−)	(−)	(−)	1x	(−)	1x
IL-6	(−)	(−)	(−)	(−)	(−)	(−)	(−)	5 ng/ml^d^	(−)	(−)

Antibiotics and antimycoplasma reagents are not listed in this table due to the wide variation in selection.

*CM* conditioned medium.

^a^Components for the culture of cervical squamous cell carcinoma organoids.

^b^Components for the culture of cervical adenocarcinoma organoids.

^c^Add these components into medium for parallel cultures.

^d^Not essential components.

^e^Components only for organoid initiation and for passaging immediately after dissociation.

Depending on the organ and whether it’s healthy or diseased, the growth factors that organoids rely on differ. Continuous growth of most female reproductive system organoids requires Wnt paracrine signaling to maintain stem cell stemness [25]. Moreover, it also needs the members of the leucine-rich repeat-containing G protein-coupled receptor (Lgr) family Lgr4, Lgr5 and Lgr6 [29–31], with the R-spondin protein family acting as Lgr receptor agonists [32]. In the culture of fallopian tube organoids, adding Wnt3a and R-spondin1 activates the Wnt signaling pathway for amplification, while Notch signaling maintains cell stemness [33]. Conversely, fallopian tube organoids with triple knockdown of *p53*, *PTEN* and *RB* which are major known tumor drivers to model high-grade serous ovarian cancer (HGSOC) development require a low Wnt environment for long-term growth [34], indicating that the signaling pathways relied upon by normal epithelium and tumor organoids may be different. Compared to healthy fallopian tube organoids that rely on BMP inhibition by Noggin, organoids from HGSOC originating from the fallopian tubes almost always require active BMP signaling [34]. Altering the types and ratios of factors in the culture medium for tumor organoids can also prevent contamination from normal cells [35, 36].

### Application scenarios of PDOs

Since organoids are derived from stem cells, the verification of organoid formation can also be used to identify stem cells, thereby assisting in the study of organ development. The endometrium continuously undergoes cycles of proliferation, secretion and shedding during the menstrual cycle. Understanding its regeneration process is of great significance for research in gynecology and regenerative biology. In the endometrium, EPCAM^+^GFP^high^Lgr5 cells [37] or long-lived bipotent epithelial progenitors expressing the Wnt reporter gene *Axin2* have been shown to grow into complete endometrial organoids [38].

As PDOs can capture patient heterogeneity, they can be applied to disease modeling, etiological and heterogeneity studies among other fields. For example, gene knockout in fallopian tube organoids can effectively characterize the origin and progression of ovarian cancer [39]. Single-cell transcriptomic analysis results once again demonstrate that key cell subgroups of the patient’s tissues can be preserved and amplified during culture, retaining heterogeneity [40].

In terms of drug testing and personalized treatment, female reproductive system tumor organoids help to deepen the understanding of tumor pathogenesis and biological features, thereby discovering new therapeutic targets and strategies. Combining high-throughput drug screening with personalized medicine technology accelerates new drug development. By establishing patient-specific organoid models, the best treatment plan can be tailored for the patient based on individual pathological characteristics and drug responsiveness. Organoids from ovarian cancer patients summarized the response to carboplatin/paclitaxel combination therapy and identified sensitive drug for 88% of patients, proving their value in drug screening [41]. Sensitivity or resistance exhibited by ovarian cancer PDOs with homologous recombination repair (HRR) deficiency to PARPi not only reflected the variability in patient drug responses but also promoted research into potential resistance mechanisms related to replication fork protection and restoration of HRR functions [42]. Moreover, for drugs with complex anti-tumor mechanisms, such as traditional Chinese medicines [43–46], organoids may offer significant advantages in studying their anti-tumor mechanisms, as they can partially recapitulate the tumor microenvironment.

## The role of organoids in female reproductive system organs and tumors

**Fig. 1:**
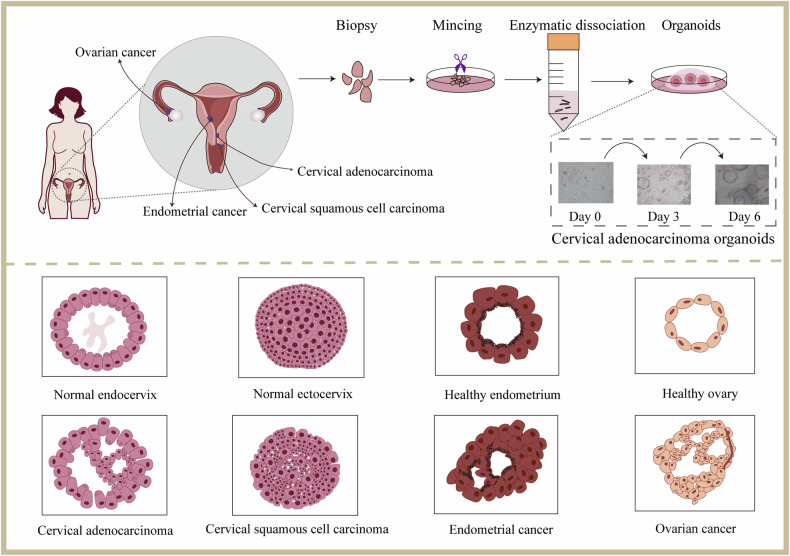
Different organoids of the female reproductive tract derived from normal or tumor tissue. Isolated or reprogrammed stem cells were cultured in an ECM surrounded by a culture medium supplemented with niche factors specific to organoids. These stem cells proliferate in the culture medium and self-organize into functional 3D structures. The brightfield images shows the morphology of cervical adenocarcinoma organoids on day 0, day 3 and day 6. Cervical adenocarcinoma organoids show a more dense structure and marked vacuolation than normal cystic endocervical organoids. The organoid structure of cervical squamous cell carcinoma is less defined, with stratified loss and poor cell polarity. Endometrial carcinoma organoids usually appear as glandular-like morphology with a well-defined to moderately defined lumen, but with higher cancer stages the structure appears dense without lumen. Ovarian cancer organoids show extensive morphological differences among different histological subgroups. Most ovarian cancer organoids have a dense structure and contain multiple lumens. The structures of these organoids were mapped based on HE staining features.

At present, organoids including cervix, endometrium, ovary and fallopian tube and their related cancers have been established and applied to drug high-throughput screening, drug response prediction, disease target screening and so on. Table 2 shows the application details of different organoid models derived from female reproductive tract organs and cancers. Different organoid models related to the female reproductive tract are also showed in Fig. 1.

**Table 2:** Organoids models for organs and cancers of female reproductive tract.

Organoid model types		Organoid sources	Numbers of organoid lines	Hotspot mutations	Model validation method	Applications	Ref.
Cervix uterus	SqCa	Patient tumor tissue	11	*TP53, ARID1B, CDKN2A, ELF3, FAT1, BRCA1/2, ATM, FANCA*	Histological analysis, phenotype markers, gene expression patterns, genetic mutations, viral transcriptome	Drug screening, xenograft, viral infection model, HPV infection study	[50]
	AdCa	Patient tumor tissue	1	*FBXW7, CASP8, NOTCH3, PDGFRB, TSC2, EPHA2, EPHA5*			
	Invasive SqCa, AdCa, LCNEC, villoglandular AdCa, SCNEC	Patient tumor tissue	17	*KRAS, TP53, KMT2C, PRSS1*	Histological analysis, diagnostic marker, copy number variation	High-throughput drug screening, radiotherapy response prediction	[59]
	HSIL	Patient HSIL tissue	22	*MUC16, PRUNE2*	Histological analysis, phenotype markers, karyotype identification, subcellular structure, HPV testing, gene expression patterns, genetic mutations	Drug screening, xenograft, co-culture with PBMC	[63]
	SqCa	Patient tumor tissue	15	*HLA-B, ERBB3, ERBB2, NDC80, KLF6, EPS8, WASF2, MARK2, PIK3R4, TRIP12, CDKN2AIP, ISG15, FLT1, UBE3A, SERPINB2, RBBP8*			
	SqCa, AdCa	Patient tumor tissue	67	*PIK3CA, EP300, FBXW7, PTEN, ARID1A, TTN*	Pathological analysis, HPV testing, phenotype markers, gene expression patterns, genetic mutations	Radiotherapy and combined platinum sensitivity testing, paired TILs killing assay, xenograft	[69]
	CCC	Patient tumor tissue	1	*PIK3C2B, MLH1, FLT1, SMARCA4, TFE3*	Pathological analysis, genomic DNA analysis	Drug screening	[65]
	SCCC	Patient tumor tissue	1	*AHNAK, CNOT1, KIAA1551, KRAS, MAP2K3, SON, XIRP2, ATP2B3*	Histological analysis, phenotype markers, genetic mutations, HPV18 integration sites	KRAS pathway inhibitor testing, xenograft	[67]
	Healthy ectocervix	Human cervix tissue	N/A	N/A	Histological analysis, phenotype markers, transcriptomic analysis, live-cell imaging, HPV testing	HPV and Chlamydia trachomatis infection study, co-culture with γδ T cells	[53, 54, 64]
Endometrium	EC	Patient tumor tissue	N/A	*FBXW7, ARID1A, PTEN, CTCF, PIK3CA, TP53, POLE, FAT1, CTNNB1*	Mutational landscape analysis	Tumor heterogeneity study, drug screening, xenograft	[9]
						Lentiviral transduction, searching therapeutic targets	[87]
	EC	Patient tumor tissue	15	N/A	Histological analysis, phenotype markers	Drug screening, response prediction to estrogen and progesterone	[82]
	EC	Patient tumor tissue	3	*PTEN, ZNF286A, ARAID1A, PIK3CA, FGF*	Histological analysis, phenotype markers, genetic mutations	Treated by cancer-associated fibroblasts culture supernatant	[90]
	EC	Patient tumor tissue	11	N/A	Pathological analysis	Drug testing and mechanism research	[83]
	EC	Patient tumor tissue	N/A	N/A	Phenotype markers	Drug testing and mechanism research	[80]
	EC	Patient tumor tissue	2	N/A	Phenotype markers	Drug sensitivity testing	[84]
	EC, serous, CCC, mixed serous and EC	Patient tumor tissue or g ascites fluid	22	N/A	N/A	Drug screening	[81]
	EMC	Patient tumor tissue	5	*PTEN, PIK3CA, ARID1A, CHEK2*	Pathological analysis, genetic mutations	Tumorigenicity study, drug screening, xenograft	[24]
Ovary	HGSC, LGSC, CCC	Patient tumor tissue or g ascites fluid	21	N/A	N/A	Drug screening	[81]
	BBT, HGSC, EMC, SBT, MC	Patient tumor tissue	9	*NF1, TP53, PKHD1, DST*	Pathological analysis, genetic mutations	Tumorigenicity study, drug screening, xenograft	[24]
	HGSC, EMC, MBT, CCC	Patient tumor tissue	18	*BRCA1, BRCA2, MLH1, PIK3CA, TP53, ARID1A, MSH2*	Histological analysis, phenotype markers, genetic mutations, copy number variations	Drug screening	[121]
	HGSC	Patient tumor tissue	6	*TP53, FANCC, NOTCH2, BRCA1, BRIP1, RAD21, ERCC4, MSH3, ABL1, MGMT*	Histological analysis, gene expression patterns, genetic mutations	Drug sensitivity testing	[123]
	HGSC	Patient tumor tissue	6	*ABL1, AKT2, ALK, ALOX12B, CHEK2, CSF3R, DICER1, EP300, ERCC4, FAT1*	Histological analysis, phenotype markers, genetic mutations,	Drug sensitivity testing, potential target searching	[125]
	LGSC	Patient tumor tissue	1	*CHEK2, AKT1, IRS2, MSH3, MUTYH, NOTCH3, MLL2/KMT2D*	Genetic mutations	Drug sensitivity testing	[97]
	MBT, SBT, MC, LGSC, CCC, EMC, HGSC	Patient tumor tissue	56	*KRAS, BRAF, TP53, MYC, CCNE1, RB1, PTEN, CDKN2A/B*	Histological analysis, phenotype markers, genomic landscape	Genetic manipulation, drug screening, xenograft, drug sensitivity testing	[39]
	HGSC, LGSC, HG, SBT, MBT, MC, EMC, CCC	Patient tumor tissue	36	N/A	Histological analysis, genomic features	Drug screening, tumor heterogeneity study	[41]
	HGSC, high-grade mixed type carcinoma, CS, HGPC	Patient tumor tissue	4	N/A	Histological analysis	High-throughput drug screening, xenograft	[131]
	HGSC	Frozen tissue from patient tumor and ascites drainage	10	*CCNE1, KRAS, MYC, MECOM, RB1, CSMD3, CDK12, KMT2B, KMT2C, CCNA2*	Histological analysis, phenotype markers, genomic landscape, genetic mutations, transcriptomic features	Drug sensitivity testing	[134]
	HGSC	Patient tumor tissue	13	N/A	Immune cell type analysis	Co-culture with immune cells, immune checkpoint blockade testing and mechanism research	[127]
	HGSC	Patient tumor tissue	33	*BRCA1, BRCA2, TP53*	Histological analysis, phenotype markers, genetic mutations	Drug screening, DNA repair profiling and therapeutic sensitivity testing	[119]
Fallopian tube	Early-stage carcinogenesis	iPSC	3	*BRCA1*	Histological analysis, differentiation markers, tumor markers, precancerous molecular properties, genetic mutations	Neoplastic conversion testing, xenograft, PARPi effect testing	[18]

This table lists the female reproductive tumor organoids that have been reported in the literature and the organoid models that can be used to mimic tumors by infection or gene editing. We selected organoids derived from human stem cells, including as many valuable studies as possible. Studies that only attempted organoid construction without sufficient characterization, representational validation, and application research are not included in this table. For the gene mutation column, if the number of mutated genes mentioned in the references is too large, the top 10 mutated genes are prioritized for display.

*AdCa* cervical adenocarcinoma, *SqCa* cervical squamous carcinoma, *LCNEC* large cell neuroendocrine carcinoma, *SCNEC* small cell neuroendocrine carcinoma, *HSIL* high-grade squamous intraepithelial lesions, *SCCC* small cell carcinoma of the uterine cervix, *EC* endometrial cancer, *EMC* endometrioid carcinoma, *CCC* clear cell carcinoma, *BBT* borderline Brenner tumor, *HGSC* high-grade serous carcinoma, *MB* malignant Brenner tumor, *MBT* mucinous borderline tumors, *SBT* serous borderline tumor, *MC* mucinous carcinoma, *LGSC* low-grade serous carcinoma, *HG* high-grade adenocarcinoma, *CS* carcinosarcoma, *HGPC* high-grade peritoneal carcinoma.

### Cervix and cervical cancer

**Fig. 2:**
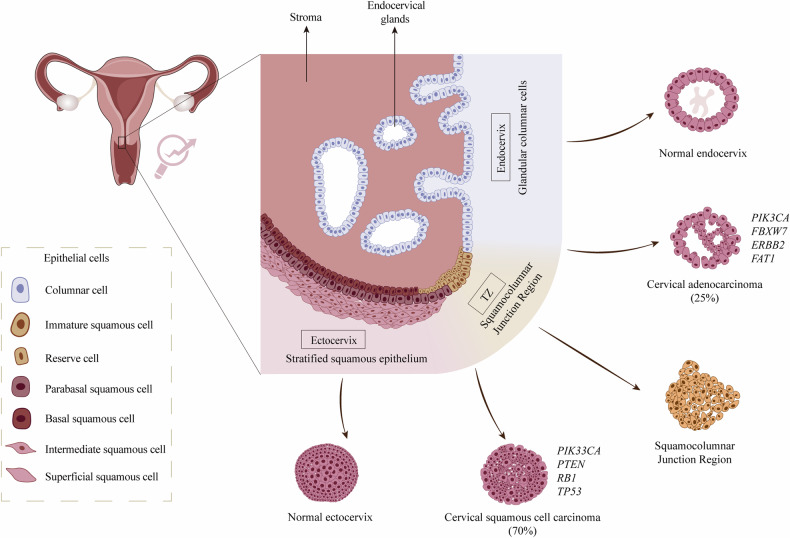
Types of cervical organoids derived from different epithelial origin using normal or tumor tissue. The cervix is composed of three parts from the position near the uterus downwards: the endocervix consisting of a single layer of columnar epithelium, the transformation zone where the columnar and squamous epithelia meet, and the ectocervix with multiple layers of squamous cells. Cervical cancer mainly includes cervical adenocarcinoma (25%) or squamous cell carcinoma (70%), while the rest are rare types such as adenosquamous carcinoma. Adenocarcinoma is characterized by mutations in the *PIK3CA*, *ERBB2*, *FBXW7* and *FAT1* genes. The mutations in *TP53*, *RB1*, *PIK3CA* and *PTEN* genes are common in squamous cell carcinoma. Organoids derived from the squamocolumnar junction usually appear round in shape, with dense and cystic characteristics, but grow irregularly after several passes, with multiple budding or chain-like structures.

The cervix is a narrow channel that connects the uterus and the vagina, performing functions such as secreting mucus and protecting the upper reproductive tract. It is primarily composed of the endocervix with its secretory columnar epithelium and the ectocervix with its multilayered squamous epithelial cells. The transformation zone between these two, where the squamous and columnar epithelia meet, is the most common area for cervical cancer to arise [47]. The epithelial structures of the cervix and the organoids derived from different epithelial origin using normal or tumor tissue are shown in Fig. 2. As a gateway to the upper reproductive tract, the cervix encounters a multitude of pathogens. Although the cervix can clear most pathogenic microorganisms and prevent ascending infections, some viruses and bacteria can evade innate immunity, leading to infections and lesions such as cervicitis, polyps, warts and cervical cancer. Cervical cancer is primarily caused by high-risk types of HPV and Chlamydia trachomatis infections. Despite the containment of cervical cancer incidence rates with the advancement of HPV testing and vaccination, GLOBOCAN2020 statistics indicated that it remained the fourth highest cause of cancer incidence and mortality among women worldwide [48].

Originally, patient-derived cervical tissue organoids were cultivated from cervical keratinocytes isolated from biopsied tissue using skin 3D organotypic rafts, but the success rate was low [49]. In 2021, the team of Hans Clevers successfully established organoids from clinically sourced healthy ecto- and endocervical epithelia. Furthermore, they constructed squamous and adenocarcinoma organoids of the cervix using small samples from pap tests and investigated the effects of HPV virus on cervical carcinogenesis. They demonstrated that cervical organoids could be cultured long-term and consistently represented the genetic mutation characteristics and tumor marker features of clinical tissues [50].

The cervical transformation zone containing the squamocolumnar junction (SCJ) is the prevalent site for HPV infection and cervical cancer [51]. The reason for the predisposition of transformation zone to malignant transformation remains unclear, but some potential hypotheses include localized immune suppression [52, 53], accelerated cell proliferation and unstable differentiation [54], increased estrogen and progesterone receptors [55], etc. Considering the differentiation observed in organotypic culture, HPV16-immortalized cells from the transformation zone or endocervix are more dysplastic, express higher levels of Ki-67 and p-AKT, and more frequently invade the collagen rafts composed of cervical stromal cells while expressing MMP-1—potentially facilitating tumor development [56]. Organoids from the cervical SCJ [11] and may offer new insights into the cytological characteristics of the cervical transformation zone and the development of cervical cancer.

Human and murine cervix organoids can be used to simulate HPV infection or Chlamydia trachomatis infection by transducing HPV oncogenes to cervical stem cells [57]. Studies on patient-derived ectocervical organoids simulating solitary and concurrent infections of HPV and Chlamydia trachomatis showed that HPV interfered with the growth of Chlamydia trachomatis leading to persistent infection. Chlamydia trachomatis infection disrupted some of the cellular protective mechanisms induced during HPV infection, including mismatch repair, increasing the likelihood of mutations and more severe cytopathic changes, thus raising the cancer development risk [58]. Besides HPV oncogenes *E6* and *E7*, introduction of *c-MYC* and *HRAS* genes can collaboratively induce cervical tumor models [59]. Stimulating the expression of putative differentiation factors and/or reducing the *SMAD4* gene can simulate the carcinogenic process of cervical adenocarcinoma in organoids. *FOXA2* was found to potentially play a significant role in regulating the histopathology and formation of cervical cancer organoids [60]. High-grade squamous intraepithelial lesions (HSIL) caused by persistent high-risk HPV infection are considered precancerous conditions of the cervix. If left untreated, HSIL can progress to cervical cancer [61, 62]. Organoids derived from patients’ HSIL can accurately represent the histological and genomic characteristics of the patient’s precancerous lesions, including HPV viral transcripts. Additionally, these organoids exhibit relative high sensitivity to standard chemotherapeutic drugs [63].

Cervical cancer PDOs could be used for drug screening and radiosensitivity testing, and serve as a platform for personalized medicine. Subjecting organoids from four different cervical cancer subtypes to screening with over 170 drugs revealed differential sensitivity in the organoids to seven of these treatments. [64]. For cervical squamous cell carcinoma, PDOs can demonstrate patient-specific drug responses to four standard chemotherapeutic compounds, including cisplatin, carboplatin, gemcitabine, and olaparib. For instance, PDOs exhibiting the highest resistance to platinum-based drugs correspondingly show consistent low sensitivity treatment outcomes in their originating patients [63].

Investigating the infiltration and cytotoxicity of immune cells towards cervical cancer cells in three-dimensional in vitro models has seen preliminary progress with spheroid models [65–67]. Recently, the development of more valuable cervical cancer organoid-immune cell co-culture models has gained attention. When co-cultured with γδ T cells, cervical cancer PDOs and HPV E6E7-transformed cervical organoids exhibited greater sensitivity to cytotoxicity compared to healthy cervical organoids. This co-culture system can be combined with RNA-seq to further explore potential immune effector pathways [68], suggesting that organoid-immune cell co-culture systems hold promise for research in immunotherapy and immune mechanisms. A recent study supports this outlook: by co-culturing PDOs with peripheral blood immune cells (PBMCs) activated by HPV associated antigenic peptides from healthy donors, immune cell-mediated structural disruption and cytotoxicity towards PDOs were observed. Moreover, PDOs identified different antigenic peptides and reacted accordingly [63]. When the immune cells were selected as tumor-infiltrating lymphocytes (TILs) matched to the patient, the PDO-TIL co-culture system showed distinct TIL-mediated tumor killing and immune responses. This co-culture model could potentially serve as a platform for simulating personalized responses to immunotherapies, such as adoptive T cell therapy [69].

Moreover, organoids of specific cervical pathological types have been successfully constructed. An organoid model of cervical clear cell carcinoma (cCCC) was successfully developed from a patient biopsy sample and xenograft models can be established from these organoids at early culture stages. This organoid closely mirrored the patient tumor in terms of histology, pathology, and genomic characteristics [70]. Organoids of small cell neuroendocrine carcinoma [71] and small cell carcinoma of the uterine cervix of clinical origin [72] have been used to evaluate sensitivity to clinical chemotherapy drugs. When combined with whole exome sequencing and RNA-seq analyses, they can assist in identifying treatment targets for specific tumor types.

### Endometrium and endometrial cancer

The endometrium is the mucosal lining of the uterus, playing a crucial role in maintaining normal female reproductive functions. The endometrium can be divided into the superficial functionalis and the deeper basalis [73]. The superficial layer is influenced by estrogen and progesterone, undergoing cyclical changes and periodic shedding during the menstrual cycle. Endometrial epithelial stem cells in the basalis drive the regeneration of the endometrium [74–76]. Dysfunction of the endometrium can lead to several common gynecological diseases, including abnormal uterine bleeding, infertility, miscarriage, pre-eclampsia, endometriosis and endometrial cancer.

Tissue from endometrial biopsies, shed menstrual endometrium and even cryopreserved tissue can be used for organoid culture [77]. In addition to histological and genetic consistency, organoids summarize the functions of endometrial tissue by responding to the administration of sex hormones, prolactin, and placental hormones and by having secretory functions. It can be said that hormonal interventions can aid organoids in recapitulating the menstrual cycle at morphological and molecular levels [78]. Endometrial organoids have demonstrated clonogenic capacity and can differentiate towards secretory glandular and ciliated phenotypes [79]. Single-cell and spatial transcriptomics have been used to compare the cell composition and gene expression of endometrial organoids with in vivo endometrium, identifying Wnt and Notch signaling as key factors regulating secretory and ciliated cell differentiation [25]. Within the organoids, Wnt-related genes *FOXJ1* and *LGR5* are highly expressed in luminal parts, while transcription factors induced by Wnt inhibition and Notch activation are highly expressed in glandular regions [25]. The varied organoid phenotypes, either cystic or compact, can be induced by different intensities of Wnt signaling [78]. Experiments indicated that estrogen induction and Notch signaling elicited differentiation of ciliated cells in endometrial organoids [80]. Intervention with Wnt and Notch signals in organoids confirmed that ciliated differentiation strongly depended on Wnt signals, while conditions with Wnt inhibition and Notch activation facilitated secretory differentiation, in which estrogen and progesterone also play crucial regulatory roles [25].

The regeneration of the endometrium after its cyclic shedding has long been a focus of stem cell and gynecological research. The *Axin2* gene was identified in organoid models as a marker of long-lived bipotent epithelial progenitor cells in the endometrial glands. Eliminating *Axin2* cells severely impacted endometrial homeostasis and regenerative capacity and might even lead to tumorigenesis [38].

Endometrial cancer is a malignant tumor whose incidence and mortality rates are rising annually [81], causing significant harm to patients’ reproductive and overall health. Patient-derived endometrial organoids have been established from conditions including endometriosis, endometrial cancer, endometrial hyperplasia, and Lynch syndrome [9]. Organoids from endometrial cancer are typically derived from tissue obtained from hysterectomy patients, suspended in expansion medium after enzymatic digestion [79, 82]. After verifying histology, mutation spectrum and tumor heterogeneity, endometrial cancer organoids consistently maintained characteristics of the originating tumors [24] and showed differential responses to various anti-tumor compounds and hormone inhibitors [82]. Combining organoids with proteomics can provide new insights into the heterogeneity of endometrial cancer [83]. Like other patient-derived tumor organoids, endometrial cancer organoids can also be used for testing potential treatment methods and for screening patient drug sensitivities [24, 82, 84, 85]. A study involving 43 endometrial cancer and ovarian cancer PDOs has demonstrated that this drug sensitivity screening can predict patient resistance to some extent [86]. Additionally, endometrial cancer organoids can be utilized to identify new therapeutic targets for endometrial cancer [87], study mechanisms of platinum-based drug resistance [88], and investigate sensitivity to death receptor ligand TRAIL therapy [89]. Single-cell sequencing of endometrial organoids and corresponding tumors revealed that ciliated cell markers (DYDC2, CTH, FOXJ1, and p73) and secretory cell markers MPST are expressed in endometrial tumors and positively correlated with disease-specific survival and overall survival rates in patients with endometrial cancer [90]. FOXA2 was also identified as a significant tumor suppressor in endometrial cancer through validation in organoid-based models, with a synergistic interaction with the PI3K signaling pathway [91]. Considering the potential of inhibition of the PI3K pathway in anti-inflammatory and anti-tumor, it would be useful to develop its inhibitors for endometrial cancer treatment [92, 93].

While various stages and grades of endometrial cancer organoids have been established, the efficiency of creating organoids from malignant tumors remains low [78]. Co-culturing cancer-associated fibroblasts isolated from endometrial cancer with endometrial cancer organoids can improve the slow growth and limited proliferation of these organoids [94]. Katcher et al. managed to establish organoids representing all histological subtypes of endometrial cancer that could be cultured long-term after refining the cultivation methods [95]. In addition to endometrial cancer, exposing a novel multicellular, scaffold-free endometrial organoid to high levels of androgens simulates the impact of elevated androgen levels on the endometrium as seen in polycystic ovary syndrome [96]. Endometriosis can also be modeled in vitro through the generation of spheroids [97]. Recently, decellularized hydrogels as novel organoid scaffolds offer new possibilities for the application of endometrial and tumoral organoids. Organoids grown in endometrial hydrogels are more similar in proteomics to native tissue compared to those cultured in Matrigel [98].

### Fallopian tube, ovary and ovarian cancer

The ovary is a female gonadal organ that produces ova and sex hormones. Ovarian cancer is a highly heterogeneous group of tumors with a lack of early symptoms and signs, often leading to its diagnosis at late stages [99]. It exhibits high drug resistance and recurrence rates, and carries a high mortality rate [100]. In 2019, the group led by Hans Clevers [39] established 56 ovarian cancer organoid lines from 32 patients, representing all subtypes of ovarian cancer, maintaining their heterogeneity. After drug screening and xenografting, it has been proved that these organoids can be used to test drug sensitivity in vitro and in vivo, and response to chemotherapy and drug resistance across different tumor subtypes. This extensive and long-term ovarian cancer organoid platform construction provided guidance for subsequent in vitro and in vivo expansion and analysis of ovarian cancer organoids. Additionally, 23 whole-genome characterized PDOs from 36 ovarian cancer patients preserved the genomic features of the original tumors and provided an overview of patient response to neoadjuvant carboplatin/paclitaxel combination therapy [41]. Compared to single-layer cells, organoids display more diverse drug responses and can reveal the correlation between drug sensitivity and DNA repair defects. Clinically determined treatment choices made based on drug sensitivity testing from organoids have brought significant clinical turning points for patients [101]. Furthermore, constructing a normal ovarian organoid alongside the cancerous organoid from the same patient allows for testing the non-specific cytotoxicity of drugs on normal cells at the tumor-killing dosages, providing more compelling evidence for evaluating drug safety [86]. As for drug resistance mechanism research, organoids have significant advantages over cell and animal models because they are closer to the in vivo environment, simulate tumor heterogeneity, and provide more realistic drug penetration and metabolism models.

HGSOC is one of the deadliest types of ovarian epithelial cancers whose origin is still uncertain. Currently, it is mainly believed that HGSOC originates from ovarian surface epithelium (OSE) and fallopian tube epithelium (FTE) [102–105]. OSE and FTE organoids from genetically engineered mice demonstrated that both can cause HGSOC with different latency and metastasis properties, possibly representing two different subtypes, and exhibiting varying chemotherapeutic drug sensitivity [106]. Human FTE organoids provide a valuable model for studying the origins and pathogenesis of HGSOC. A large number of stem cells have been found in the FTE cells, which can effectively form spheroids and organoids in a Wnt environment [107, 108], and organoids can respond to progesterone and estradiol [33]. The fallopian tube is a conduit for oocyte, it is a muscular tube lined by simple columnar epithelium containing secretory and ciliated cells, which produce tubular fluid and facilitate transport of gametes, respectively [109, 110]. Different Wnt and Notch signals can stimulate cilia and secretory cell differentiation in fallopian tube organoids [33], which is significant for understanding fallopian tube lesions and carcinogenesis. The secretory cells of the fallopian tube epithelium are the origin cells of serous tubal intraepithelial carcinoma (STIC), which is considered a precursor to HGSOC [111, 112]. In recent years, abnormal proliferation of secretory cells, often referred to as early serous proliferations (ESPs), has been suspected to be a direct precursor to HGSOC [113, 114], and this process is often associated with *TP53* mutations [115]. HR deficiency caused by *BRCA1/2* mutations is considered another important risk factor for the occurrence of HGSOC [116]. Fallopian tube organoids derived from iPSCs of ovarian cancer patients carrying *BRCA1* mutations showed cell abnormalities consistent with tumor development [18]. Similarly derived from iPSCs, FTE organoids from healthy donors are established through differentiation steps, such as Mullerian duct and fallopian tube epithelial precursors. These organoids encompass cell types representing the mature differentiation of the FTE lineage and can self-organize into lumen-forming structures. These iPSC-derived FTE organoids provide a powerful in vitro model for studying FTE cell transformation and the early development of HGSOC. Furthermore, using Chlamydia trachomatis to infect fallopian tube organoids enhanced cell stemness and accelerated DNA aging. This suggests that chronic infection of FTE by Chlamydia trachomatis may be a potential factor in fallopian tube lesions and the development of HGSOC [117].

Since mouse-source tumor tissue is easily obtainable and shows similarity to the human HGSOC model in genetic dependence surrounding the tumor microenvironment and drug response [118], it has applied advantages. Constructing HGSOC of homologous recombination (HR)-proficient (*Trp53*^*−/−*^; *Ccne1*^*OE*^; *Akt2*^*OE*^; *Kras*^*OE*^), HR-deficient (*Trp53*^*−/−*^; *Brca*^*1−/−*^; *Myc*^*OE*^), and unclassified (*Trp53*^*−/−*^; *Pten*^*−/−*^; *Nf1*^*−/−*^) organoids from mice with genetic engineering source provided fast evaluation platforms for developing effective treatment methods, resolving the difficulty in treating the *CCNE1* subtype that is highly drug-resistant. These models reveal genotype-specific immune microenvironments and chemotherapeutic sensitivities, demonstrating durable T-cell dependent responses in HR-proficient genotypes and none in others, underscoring the significance of immune context in therapeutic development [118].

Organoids established from clinical tumor samples better represent tumor heterogeneity. After bridging species differences, they could be used more accurately for drug screening, including targeted drugs and immune therapy. Based on a fast-growing and high success rate HGSOC organoid platform, it proved that organoids’ sensitivity to PARPi was only related to HR dysfunction but not DNA repair gene mutation status, while the sensitivity to paraplatin, CHK1, and ATR inhibitors related to replication fork protection deficiency [119]. Since the launch of PARPi for the treatment of ovarian cancer, it has revolutionized the treatment of the disease by targeting HR deficiency, which accounts for approximately 41–50% of ovarian cancer [120, 121]. HR deficiency caused by BRCA1/2 mutations is considered another important risk factor for the occurrence of HGSOC. Similar to other genetic factors, HR deficiency is associated with cancer susceptibility and prognosis [122, 123]. In drug testing facing 23 ovarian cancer PDOs, all PDOs showed resistance to olaparib, rucaparib, and niraparib, consistent with HR deficiency classification based on whole-genome sequencing data [41]. The PARPi exhibit synthetic lethality to selectively kill tumor cells with HR deficiency, with *BRCA1/2* mutations being the most significant pathogenic mutations in HRR related-genes [122, 124]. This was confirmed in organoids carried *BRCA1* mutations, which exhibited a higher sensitivity to the PARPi olaparib and platinum-based drugs [125]. However, exceptions exist where resistance to PARPi occurs despite *BRCA2* deletions [126]. Combining with patients’ clinical drug responses, HGSOC PDOs derived from debulking surgeries (two neoadjuvant-carboplatin-exposed and four chemo-naïve) have been demonstrated to be predictive of carboplatin resistance [127]. This platinum drug resistance may be associated with RAD51 expression levels, which has been observed and validated in both HGSOC organoids and patient cohorts. A low RAD51 score predicts sensitivity to platinum-based drugs, as well as better progression-free survival and overall survival [128]. Moreover, by testing the effects of inhibitors and analyzing transcriptomic changes, patient-derived HGSOC organoids can also be used to identify potential therapeutic vulnerabilities [129].

The interaction between the tumor immune microenvironment and the tumor itself is often difficult for organoids to replicate, yet it is crucial. Tumor-derived UBR5 has been shown to promote the recruitment and activation of tumor-associated macrophages, leading to ovarian cancer progression. Additionally, UBR5 promotes organoid formation by controlling p53 protein levels through β-catenin-mediated signaling [130]. By incorporating immune cells into the organoid culture system, the co-culture model of ovarian cancer organoids and immune cells has provided valuable insights for developing ovarian cancer immunotherapy and identifying new immune therapeutic targets. Testing the effects and cellular states following bispecific anti-PD-1/PD-L1 antibody treatment revealed that enhanced immunotherapy efficacy depends on the activation and cytotoxic activity of NK and CD8 T cell subsets. These cellular state changes are partially regulated by downregulation of the bromodomain-containing protein BRD1 [131], which contributes to understanding the epigenetic regulation of resistance to PD-1/PD-L1 blockade cancer immunotherapy [132]. Furthermore, 3D organotypic models of ovarian cancer containing primary human fibroblasts and mesothelial cells can be used for high-throughput screening of a wide range of antitumor drugs [133]. In the future, organoid co-culture models that include a greater variety of cell types and more closely mimic the complex tumor microenvironment will become more powerful preclinical models.

Building HGSOC organoids with higher success rates, including organoid establishment from cryopreserved tissues [134], and developing more high-throughput drug screening platforms, are some areas of exploration to expand the application of ovarian cancer organoids. Phan et al. developed a platform for the high-throughput identification of drug sensitivity for HGSOC and ovarian sarcoma, demonstrating the feasibility of using 240 kinase inhibitors for testing and achieving an excellent fit with clinical decision times in just 1 week [135].

### Vaginal cancer and vulva cancer

The vaginal epithelium can be divided into three layers based on the degree of cell differentiation: the basal layer, composed of basal cells; the intermediate layer, consisting of multiple layers of polygonal or prickle cells; and the superficial layer, made up of keratinized or nearly keratinized squamous epithelial cells [136]. The basal cells, which have a high proliferative capacity, are the proliferating region of the vaginal epithelial cells and thus have a strong ability to form organoids [137]. Notably, Axin2-labeled basal cells can self-renew without relying on hormones [137]. Similar to other female reproductive system organoids, the proliferation and differentiation of vaginal organoids are also regulated by the key Wnt signaling pathway. Even after ovarian removal, Axin2-expressing vaginal epithelial cells can respond to Wnt signaling and significantly promote vaginal epithelial regeneration following estradiol administration [137]. In addition to organoids, the human vaginal mucosa organ chip (vagina-on-a-chip) provides a valuable preclinical model for understanding interactions between the vaginal microbiome and host tissues [138]. Another commercially available in vitro 3D tissue model, MatTek EpiVaginal, can be used for studying vaginal drug delivery, pharmacokinetics, and other applications [139, 140]. Vaginal cancer and vulvar cancer are relatively rare types of tumors, accounting for about 4% of gynecological tumors [4]. Similar to cervical cancer, the main cause of vaginal and vulvar cancer is also high-risk HPV infection. However, there is currently a lack of established vaginal and vulvar cancer organoids, in the future, the use of organoid technology will better bridge the gap in preclinical research on vaginal and vulvar cancer.

## Challenges and prospects of organoids in female reproductive tract tumors

Despite the increasing maturity and widespread application of organoid technology, there are still limitations as a novel model. Tumor organoids, which largely arise from epithelial cells, often lack the blood vessels, nerves, immune cells, and fibroblasts needed to reproduce the complex tumor microenvironment. This limits their ability to model cell-cell interactions, cell-ECM interactions, and cell-medium interactions. Although significant progress has been made in co-culturing gynecological tumor organoids with immune cells, the establishment of more complex co-culture models that include other cell types is still quite limited in gynecological tumor organoid research. For example, co-culture of tumor organoids with endothelial cells can mimic angiocrine crosstalk in real tumors and can be a critical model for understanding interactions between angiogenesis and the immune environment [141]. At the same time, by integrating multiple omics data (such as genomics, transcriptomics, proteomics and metabolomics, etc.), a more comprehensive and precise understanding of the composition and changes of tumor microenvironment can be obtained.

Furthermore, co-culturing organoids with microbiota is a powerful model for studying their interactions in female reproductive tract cancer, especially in relation to endometrial and ovarian cancers [142, 143]. Currently, co-culture of organoids with gut microbiota has seen many explorations, including dispersed single-cell co-culture with bacteria [144], microinjection co-culture [145] and anaerobic bacterial co-culture [146]. If more complex organoid models including cell-microbial interactions can be constructed in the field of female reproductive tract cancer, it will provide a great boost to the accurate modeling of female reproductive tract cancer.

Gene editing techniques such as CRISPR-Cas9 have shown great potential in life science research [147, 148]. CRISPR-Cas9 gene editing has been used in various organoid models to study organ development, gene function and disease modeling [149–153]. Selectively activating or inhibiting gene expression, epigenetic regulation at specific sites, or whole-genome screening through gene editing techniques can help reveal key genes and pathways involved in tumor development and verify their function. For example, CRISPR-Cas9 introduction of Trp53, Brca1, Nf1, and Pten mutations in OSE and FTE organoids were used to confirm the origin of HGSOC [154], and TP53 and RB1 knockout was used to study their impact in patient-derived ovarian cancer organoids [39]. A specialized study investigating the application of base editors in human adult stem cell-derived cancer organoids has been conducted. Using a C > T base editor-mediated CRISPR-stop, targeted stop codons were introduced in endometrial cancer-relevant genes. By combining different cytosine base editors, endometrial tumor organoids for the study of early tumorigenesis can be generated. Additionally, simultaneously introducing oncogene activation and tumor suppressor inactivation mutations can further enhance the development of tumor models [155]. In addition to CRISPR-Cas9, a novel gene labeling method called In-trans paired nicking has been developed that prompts fluorescent gene labeling of organoids without the need for double-stranded breakage [156]. Nanoblade may also be a new organoid-appropriate gene-editing technology to replace CRISPR-Cas9 engineering [157].

In addition, ECM and growth factors added during organoid culture may affect drug screening [158], and established culture conditions may be optimal for specific cancer cell subtypes. The commonly used ECM from EHS mouse sarcoma lacks identification of its components and has potential immunogenicity, which obstructs more precise disease modeling. Currently, decellularized extracellular matrix (dECM) [159, 160] and polyethylene glycol hydrogel [20] are being developed as new organoid culture matrices. Human or pig ECM-derived hydrogels have been used to support human-derived organoid culture [159, 161]. Compared to Matrigel, dECM provides the accurate microenvironment characteristics suitable for different tissue types with its special physical properties, allowing it to be used in minimally invasive delivery[162]. Polyethylene glycol hydrogel, as a synthetic matrix with clear physical and chemical parameters, has been used for the expansion of intestinal stem cells and the culture of intestinal organoids [163]. Replacing them with soft fibrin matrices also supports the culture of intestinal epithelial organoids [164]. In endometrial organoids, this fully defined synthetic polyethylene glycol hydrogel matrix can be used to study epithelial-matrix crosstalk in the face of endometrial inflammation [165]. Overall, the development of increasingly specific and clear composition new matrices will provide new support for the further applications of organoids.

The organ-on-a-chip technology, based on a microfluidic platform, enables more complex and dynamic simulations of tissues and diseases using small-scale cultures. Recently, organ-on-a-chip models have been applied to the female reproductive system and even gynecological tumors (cancer-on-a-chip), encompassing nearly all major female reproductive organs as well as gynecological cancers, including cervical cancer, endometrial cancer, and ovarian cancer [166]. By culturing primary human vaginal epithelial cells on the upper surface and primary human uterine fibroblasts on the lower surface of an extracellular matrix-coated porous membrane within a microfluidic system, a human vagina chip can be constructed. Further co-culturing this model with microorganisms allows for the identification of interactions between different vaginal microbial consortia and host tissues [138]. In recent years, the advent of microfluidic technology has offered valuable models for enhancing the management of gynecological tumors. For instance, microfluidic chips can regulate oxygen gradients and nutrients in ovarian cancer, effectively simulating cell growth, migration, invasion, apoptosis, and drug response [167, 168]. These chips can be used in ovarian cancer diagnostics to screen for new biomarkers [169, 170] or to detect tumors using exosome detection techniques that utilize nanomaterials [171, 172], as well as in therapy development [173]. For cervical cancer, microfluidic platform-based detection methods [174, 175] and drug research [176] have also been developed. These cancer-on-a-chip models, due to their precise control of fluid dynamics and chemical gradients, offer promising approaches for early cancer diagnosis and have potential for evaluating therapeutic strategies. Multi-organ chips integrate multiple different organoid types on one chip, changing the current isolated use of a single tissue type in mainstream organoid research and enabling drug compound screening [177] and evaluation of prodrug metabolism and downstream toxicity of drugs [178]. The use of the polysaccharide-based synthetic hydrogel VitroGel-ORGANOID-3 to integrate dendritic cells-gastric organoids to the gut organoid flow chip (GOFlowChip) enables faster and more accurate study of immune cell-organoid interactions [179]. In the future, combining organoids of female reproductive system tumors with microfluidic platforms may enable more refined and controllable in vitro tumor simulations.

**Fig. 3:**
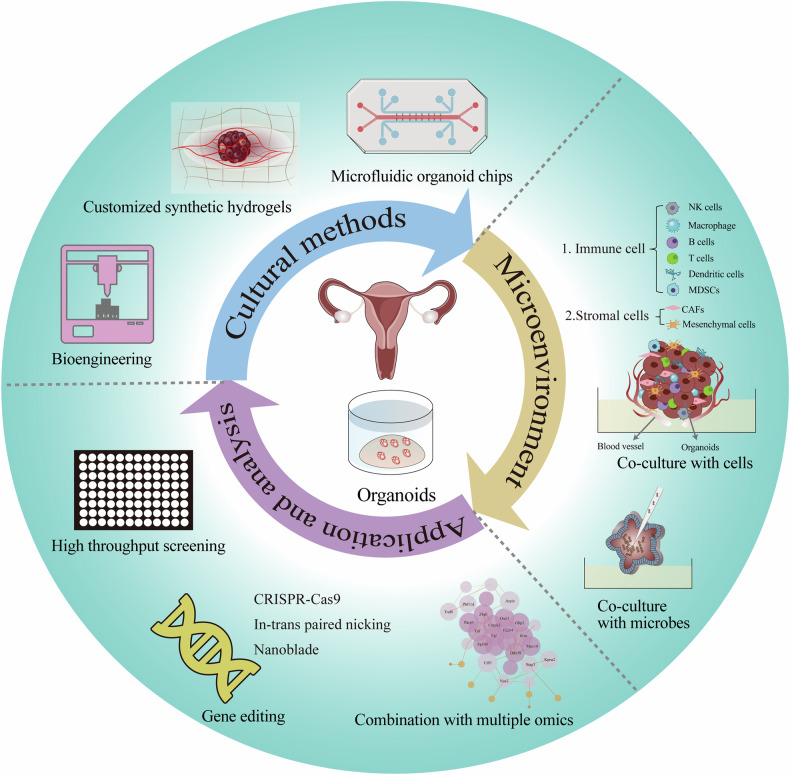
The future development of female reproductive tract organoids. In terms of culture methods, the large-scale controllable production of organoids and new culture methods can be achieved by combining bioengineering, customized synthetic hydrogels and microfluidic organoid chips. Various cells and microbes may be co-cultured in vitro to simulate the body microenvironment. At the same time, the application of organoids will be expanded by combination with multi-omics, new gene editing methods and other technologies, and it will be used in high throughput drug screening.

Moreover, improving organoid generation efficiency, reducing costs, and developing methods suitable for high-throughput drug and immune therapy screening can shorten the preclinical screening cycle and dock with the treatment window. Currently, organoid-based high-throughput screening platforms have been developed, including Z-stack imaging and fluorescent labeling to evaluate survival rate [180], miniaturization of organoids in high-density wells to suit high-throughput screening [181], and even fully automated liquid handling robots for high-throughput screening [182]. By applying microfluidics and biomaterials to organoid culture and utilizing MicroFlu-IDIC technology for precise control, dynamic physical conditions can be provided to uniformly cultivate a large number of organoids while maintaining a complex microenvironment [183]. Combined with biotechnology techniques such as tissue engineering and 3D printing, it is possible to construct more complex and realistic organoid structures in a stable and rapid manner, further improving the biological similarity and reliability of organoid models. The future development of female reproductive tract organoids is shown in Fig. 3.

## Conclusion

The female reproductive system is crucial to women’s health and reproductive function, and its diseases and even cancer pose challenges for many women worldwide. The lack of representative models hinders research on gynecologic tumors. Organoid technology, especially tumor organ models, has been increasingly applied in the study of female reproductive system tumors due to their advantages of high heterogeneity, easy accessibility and cultivation. They play a significant role in understanding the origin and causes of tumors, drug screening and personalized treatment. In the future, the combination of female reproductive system tumor organ models and various new technologies will help further understand the occurrence of female reproductive system tumors, develop new treatment methods, and open up new possibilities for personalized and precision medicine. This will contribute to improving treatment outcomes and survival rates for female reproductive system tumor patients, providing better protection for women’s health and reproductive function.

## Data Availability

The materials in this study are available to provide by corresponding authors on reasonable request.
